# Novel Method for Removing Embedded Cactus Spines in the Emergency Department

**DOI:** 10.1155/2019/6062531

**Published:** 2019-04-08

**Authors:** Andrew M. Ford, Steven T. Haywood, Douglas R. Gallo

**Affiliations:** Department of Emergency Medicine, Summa Health – Akron City Hospital, Akron, OH, USA

## Abstract

Injuries from cactus spines can present challenges to Emergency Medicine providers. When the patient has mental limitations that prevent cooperation with removal, these challenges grow. Traditional removal techniques have several drawbacks including prolonged time for complete removal and incomplete removal. We present the case of a 22-year-old with a history of low-functioning autism and congenital motor dysfunction with a cactus spine injury to a large surface area of her chest, abdomen, and extremities. Conscious sedation utilizing intramuscular ketamine and Operating Room (OR) hair removal mitts were utilized to quickly and effectively remove the cactus spines. The patient had efficient, painless resolution of her injury without need for additional spine removal.

## 1. Introduction

With the widespread availability of indoor climate control, cactus plants have become a mainstay in modern interior design across the United States. As natives of the Southwest United States, they are acclimated to dry environments making them ideal houseplants. Though visually appealing, they can also be dangerous because of their spines, which can break through skin with relatively little pressure.

We present a case where a patient fell into a cactus glochidia and received dozens of thorn injuries. We document the first successful glochidia removal using several pairs of OR hair removal mitts.

## 2. Case Report

A 22-year-old woman with a history of low-functioning autism and congenital motor dysfunction presented to the emergency department (ED) at Summa Health – Akron City Hospital (Akron, OH) with numerous cactus spine puncture wounds. Four days prior to presentation she fell into her parents' decorative* Opuntia *(e.g., “prickly pear”) cactus. The wounds were distributed throughout her torso and upper and lower extremities. There was slight erythema surrounding the embedded spines. While the patient could not cooperate or provide a history because of her nonverbal status, her pain was evident in her moans, cries, and winces as the providers touched the spines during her physical examination.

The patient was morbidly obese with limited ability to ambulate. Her parents and an aide accompanied her to the ED. They reported an extensive history of combative behavior towards healthcare providers. For this reason, conscious sedation with ketamine was initiated prior to spine removal. 4 mg/kg of intramuscular ketamine was administered. Once conscious sedation was achieved, a team of four providers removed the spines using adhesive preoperative hair removal mitts. After fifteen minutes, essentially all of the superficial needles had been removed, with the exception of a few spines that were too deep to be removed with the adhesive gloves. The patient's shirt was removed and disposed of as it too was covered in numerous spines. There was no incidence of hypoxia or emergence reaction following the administration of Ketamine.

Within the next hour, the patient recovered to her baseline mental status and was ambulating throughout the ED with her typical gait. Prior to discharge, she was given an oral dose of 875 mg amoxicillin/25 mg clavulanate (Augmentin) and an intramuscular dose of Tdap (tetanus immunization); her parents were instructed to bring her back to the ED if any fevers, chills, or swelling of the wounds occurred.

The patient was evaluated 2 weeks after the injury and was noted to have some persistent erythema on her arms and anterior thighs. The patient was given a prescription for Augmentin to be taken twice daily for 7 days. No additional spine removal was required.

Upon repeat evaluation at 4 weeks, the patient demonstrated complete resolution of the erythema and no further spine removal was necessary.

## 3. Discussion

Cacti fall under the broad category of plants known as succulents, which have become increasingly popular as ornamental plants. The cactus family is one of the largest succulents, consisting of approximately 1750 species [1]. Many cactus species feature spines or glochidia (diminutive hair-like thorns) of varying sizes [2]. These spines and glochidia are sharp and penetrate skin with minimal pressure. Our patient's wounds were caused by an* Opuntia *cactus' glochidia. These injuries are seldom amenable to conventional removal with tweezers due to their miniscule size and tendency to cause dozens of wounds at once. Furthermore, excessive force can cause them to break and migrate deeper, which can potentially lead to further complications such as granulomatous inflammation and dermatitis [2].

Current methods for removing glochidia as described in the existing literature depend on the number of spines embedded. For a single glochid, removal can be done with tweezers. However, multiple glochidia, sometimes hundreds, are typically embedded during these injuries. For injuries with multiple glochidia, the removal can be done by applying a layer of household glue over the site of injury and then peeling it away, hopefully taking the spines with it. An alternative method is to use adhesive tapes. Both of these methods have limitations. Adhesive tape has only been shown to remove 28%-30% of spines. Household glue requires 35 minutes to fully dry before it can be removed [3]. Additionally, household glue is not often available in the Emergency Department.

While these methods alone can be adequate for removal of small numbers of spines in compliant patients, they may prove insufficient in a combative patient with limited verbal capabilities. Agitated patients may complicate the wound removal process by inadvertently placing more pressure on the embedded glochidia, causing them to migrate deeper, outside of the depth at which these methods can be effective. OR hair removal mitts coupled with conscious sedation overcame this issue in our patient. We were able to treat considerable surface area in a short time, allowing our team to finish the removal prior to the cessation of conscious sedation.

The use of OR hair removal mitts for glochidia removal could prove beneficial not only in combative patients with these injuries, but also for individuals with glochidia penetrations involving a large portion of their body surface area. This technique proved to be quick and effective and is also relatively inexpensive. It can be performed by physicians, nurses, PAs, and medical students alike with minimal training.

This method is not without its shortcomings. For one, the mitts would not be sufficient to retrieve a deeply embedded glochidia or spine. For these, ultrasound-guided removal using tweezers is a well-documented approach [4]. OR mitt removal would not be ideal for addressing injuries caused by cacti with larger spines; these injuries also tend to be amenable to tweezer removal.

With the rising popularity of ornamental cacti in areas where they are not indigenous, it is important to be aware of the injuries that they may cause and how to treat them. Our case demonstrates a novel removal method that proved particularly useful for treating a widespread glochidia injury in a combative patient. This method could prove useful for treating similar injuries in other patients, be they combative or compliant. It may prove to be more efficient than other methods such as tweezers and adhesive tape.
